# Antifungal Activities of New Coumarins

**DOI:** 10.3390/molecules17055713

**Published:** 2012-05-14

**Authors:** Ahmed A. Al-Amiery, Abdul Amir Hassan Kadhum, Abu Bakar Mohamad

**Affiliations:** 1 Chemical Engineering Faculty, University of Kabengassan Malaysia (UKM), Selangor 43000, Malaysia; 2 Applied Chemistry division, Applied Science Department, University of Technology (UOT), Baghdad 10066, Iraq

**Keywords:** antifungal, 4-aminoantipyrine, benzyl bromide, coumarins, DFT, ethyl bromoacetate, 4-hydroxycoumarin

## Abstract

Newly synthesized coumarins 4-((5-mercapto-4-phenyl-4*H*-1,2,4-triazol-3-yl)-methoxy)-2*H*-chromen-2-one and 4-((5-(phenylamino)-1,3,4-thiadiazol-2-yl)-methoxy)-2*H*-chromen-2-one were tested against selected types of fungi and showed significant activities. DFT calculations of the synthesized coumarins were performed using molecular structures with optimized geometries. Molecular orbital calculations provide a detailed description of the orbitals, including spatial characteristics, nodal patterns, and the contributions of individual atoms.

## 1. Introduction

The serious medical problem of bacterial and fungal resistance and the rapid rate at which it develops has led to increasing levels of resistance to classical antibiotics [1,2,3], and the discovery and development of effective antibacterial and antifungal drugs with novel mechanisms of action have thus become urgent tasks for infectious disease research programs [4,5]. Coumarin and its derivatives represent one of the most active classes of compound possessing a wide spectrum of biological activity [6,7,8,9,10,11,12]. The interesting biological activity of these coumarins made these compounds attractive targets in organic synthesis. Several synthetic strategies for the synthesis of coumarins have already been developed. Coumarins can be synthesized by the Perkin reaction, Pechmann reaction or by Knoevenagel condensation of salicylaldehydes with malonic acid, or Meldrum’s acid [13]. malonic esters, cyanoacetic esters. Recently, the Wittig reaction in *N,N*-diethylaniline was also conveniently applied for the synthesis of coumarins [14]. The preparation of ethyl 2-(2-oxo-2*H*-chromen-4-yloxy)-acetate (**2**), which was used as a starting material for the synthesis of compounds **5** and **7**, is presented in this study. The chemical structures of the synthesized compounds **2**–**7** were determined and confirmed. The *in vitro* antifungal activities of the synthesized compounds **5** and **7** were investigated.

Structure activity relationships of coumarin derivatives have revealed that the presence of substituted amino derivatives is an essential feature of their pharmacological action. Based on these findings, we try to describe the synthesis of some compounds featuring different heterocyclic rings fused onto the coumarin moiety with the aim of obtaining more potent pharmacologically active compounds.

## 2. Results and Discussion

### 2.1. Chemistry

**Scheme 1:**
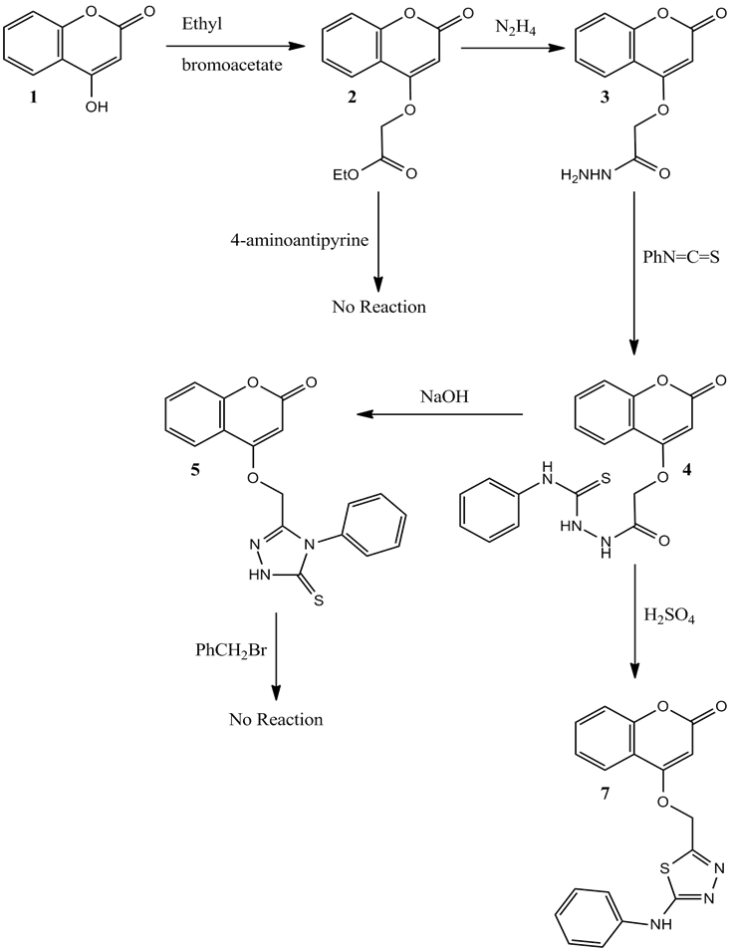
Reaction sequences of the synthesized compounds.

For the synthesis of new coumarins **2**–**7**, the reaction sequence is out lined in Scheme 1, started from 4-hydroxycoumarins **1**. 

**Scheme 2:**
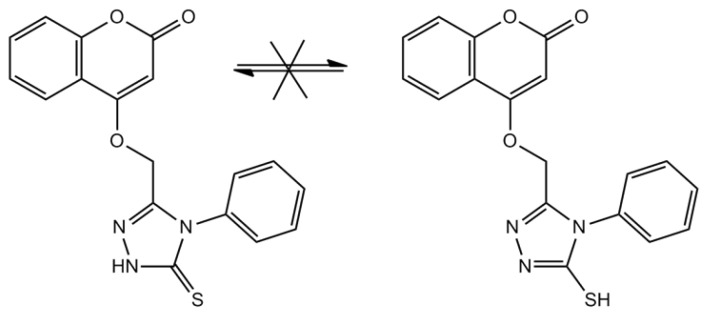
Tautomerization of compound **5**.

Compound **2**, (ethyl 2-(2-oxo-2H-chromen-4-yloxy) acetate) was obtained by the reflux of ethyl bromoacetate with 4-hydroxycoumarin (**1**) in the presence of anhydrous potassium carbonate in anhydrous acetone. Reaction of compound **2**, with various amines was very difficult, even when different solvents (methanol, ethanol and acetone) were used and only hydrazine reacted with compound **2**. Hydrazinolysis of compound **2** with hydrazine hydrate afforded hydrazide **3** in good yield. The FT-IR spectrum of compound **3** showed absorption bands in the 3297.3, 3211.0 cm^−1^ (hydrazide NH-NH_2_), 1671 cm^−1^ (lactonic-C=O carbonyl stretching), and 1,711.5 cm^−1^ (esteric-C=O carbonyl stretching). The ^1^H-NMR spectrum exhibited a singlet due to the –CO-NH-NH_2_ proton at δ 8.89 ppm. Compound **3** was allowed to react with isothiocyanatobenzene to yield compound **4** that can be cyclized either with sodium hydroxide to yield compound **5** that cannot react with benzyl bromide, or cyclized with sulphuric acid to yield compound **7**. For compound **4**, the IR spectrum has the following characteristic absorption bands: ν_N-H_ (3368.9, 3371, 3350, 3262 cm^−1^); ν_C=O_ (1760 lactone; 1686 ester cm^−1^), ν_C=S_ (1276 cm^−1^). In the IR spectrum of compound **7**, no absorption band was detected about 1700 cm^−1^ indicating the absence of the ester carbonyl group, which is evidence for the conversion of compound **4** to compound **7**. Also, in the IR spectrum of the new heterocyclic compound **7** a stretching band characteristic of the C=N group from heterocyclic nucleus was seen at 1599 cm^−1^ (from thiadiazole). Although two types of tautomers, thione or thiole (Scheme 2), could be expected from the cyclization of compound **5**, under basic media, only the thione type compound **5** was observed. Existence of the thione form predominantly in the solid state is demonstrated by the presence of two absorption bands at 1240 cm^−1^ and 3408 cm^−1^ belonging to the ν_C=S_ and ν_NH_ groups, respectively, and by absence of ν_SH_ [15]. Reaction of compound **2** with 4-aminoantipyrine was not successful, even when we use dimethyformamide as solvent, moreover no product was formed when we try to react compound **5** with benzyl bromide.

### 2.2. Computational Studies

#### 2.2.1. Atomic Charges and Stabilities

**Figure 1:**
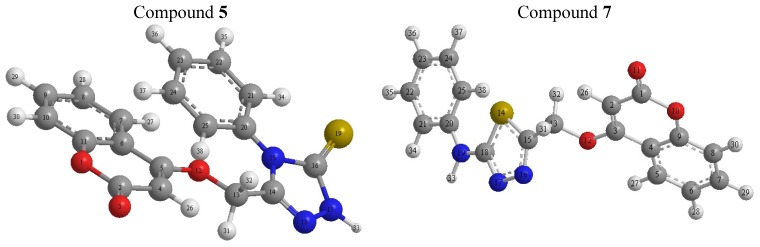
3D-geometrical structures for compounds **5** and **7**.

The theoretical studies for compound **5** revealed that the atomic charges have been affected by the presence of the ring substituent. The minimized geometry is shown in Figure 1, where the calculated atomic charges for the compounds **5** and **7** are also indicated. For compound **5** the highest atomic charge is at [O(3) −0.57)] and the next charge values are at [N(18) −0.512] and [S(19) −0.38]. These results clearly indicated that these three atoms are the most reactive sites toward the reactions and bonding with the metals. The calculated bond and twist angles and 3D-geometrical structure, indicated that this molecule is not planar moreover the stereochemistry is C(5)-C(4): (E) and N(18)-C(14): (Z). The C(13)-C(14) bond length is 1.4970; O(12)-C(13) is 1.4020 and C(5)-O(12) is 1.3550.

For compound **7** the highest atomic charge is at [O(11) −0.805)] and the next charge value is at [C(2) −0.433]. These results clearly indicated that these atoms are the most reactive sites toward the reactions and bonding with the metals. The calculated bond and twist angles and 3d-geometrical structure, indicated that this molecule is not planar moreover the stereochemistry is C(3)-C(2): (*E*). The S(14)-C(18) bond length is 1.7159; S(14)-C(15) is 1.7158; C(18)-C(19) is 1.2660 and C(20)-C(19) is 1.2660.

#### 2.2.2. Density Function Theory (DFT)

**Figure 2:**
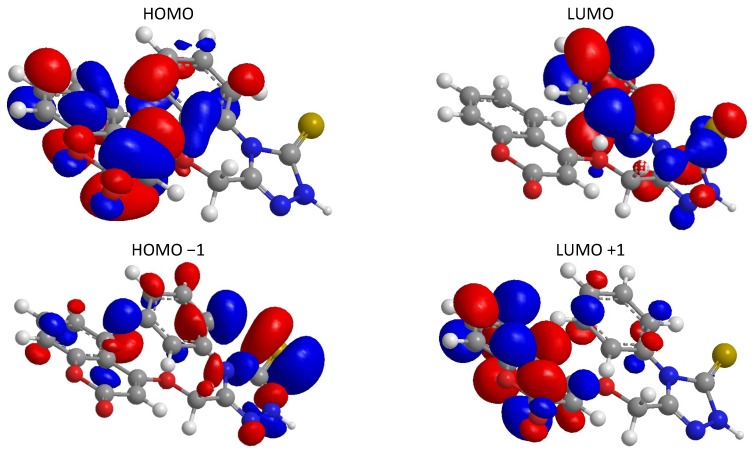
HOMO, LUMO, HOMO −1 and LUMO +1 orbitals of **5**.

**Figure 3:**
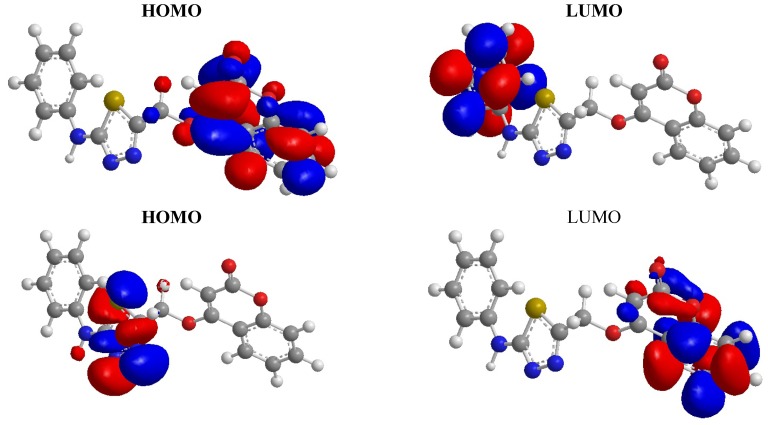
HOMO, LUMO, HOMO −1 and LUMO +1 orbitals of **7**.

DFT calculations were performed for compound **5**. The optimized molecular structures of the most stable forms are shown in Figure 1. Molecular orbital calculations provide a detailed description of orbitals including spatial characteristics, nodal patterns and individual atom contributions. The contour plots of the frontier orbitals for the ground state of **5** and **7** are shown in Figure 2 and Figure 3, including the Highest Occupied Molecular Orbital (HOMO) and the Lowest Unoccupied Molecular Orbital (LUMO). It is interesting to see that both orbitals are substantially distributed over the conjugation plane. It can be seen from Figure 2 and Figure 3 that the HOMO orbitals are located on the substituted molecule while LUMO orbitals resemble those obtained for the unsubstituted molecule and therefore the substitution has an influence on the electron donation ability, but only a small impact on electron acceptance ability [16,17,18]. The orbital energy levels of HOMO and LUMO of compound **5** and **7** are listed in Table 1. It can be seen that the energy gaps between HOMO and LUMO is about 2.241 eV. for compound **5**, and the lower value in the HOMO and LUMO energy gap explain the eventual charge transfer interaction taking place within the molecules. 

For compound **7**, the energy gap between HOMO and LUMO is about −2.570 eV. The lower value of the HOMO and LUMO energy gap explains the eventual charge transfer interaction taking place within the molecules.

**Table 1:** HOMO and LUMO energies of **5** and **7** (**eV**).

HOMO	LUMO	∆E	HOMO −1	LUMO +1	∆E
−2.840	−0.599	−2.241	−6.415	−0.205	−6.210
−3.625	−1.055	2.570	−8.830	−0.067	8.763

### 2.3. Antifungal Activity

According to Overtone’s concept of cell permeability, the lipid membrane that surrounds the cell favors the passage of only lipid-soluble materials, so lipophilicity is an important factor controlling the antifungal activity. Delocalization of π-electrons over the compounds **5** and **7** increases lipophilicity which in turn facilitates the penetration of the compounds **5** and **7** into lipid membranes, further restricting proliferation of the microorganisms. Although the exact biochemical mechanism is not completely understood, the mode of action of antimicrobials may involve various targets in the microorganisms:

• Interference with the synthesis of cellular walls, causing damage that can lead to altered cell permeability characteristics or disorganized lipoprotein arrangements, ultimately resulting in cell death.
• Deactivation of various cellular enzymes that play a vital role in the metabolic pathways of these microorganisms.
• Denaturation of one or more cellular proteins, causing the normal cellular processes to be impaired.
• Formation of a hydrogen bond through the azomethine group with the active centers of various cellular constituents, resulting in interference with normal cellular processes [19,20,21,22,23,24].

**Figure 4:**
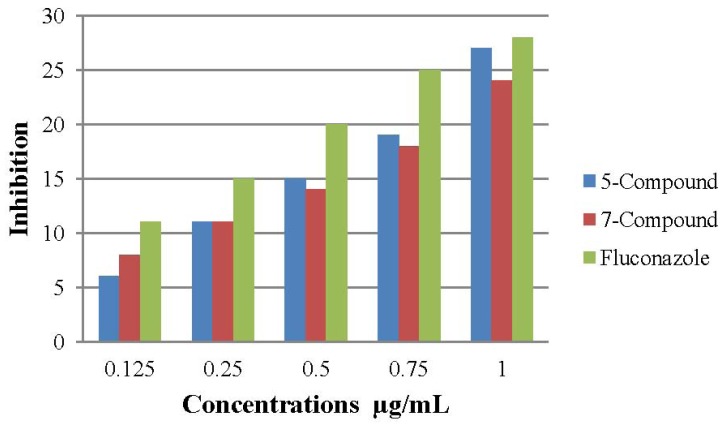
The effect of compounds **5** and **7** towards the tested organism *Aspergillus niger*.

**Figure 5:**
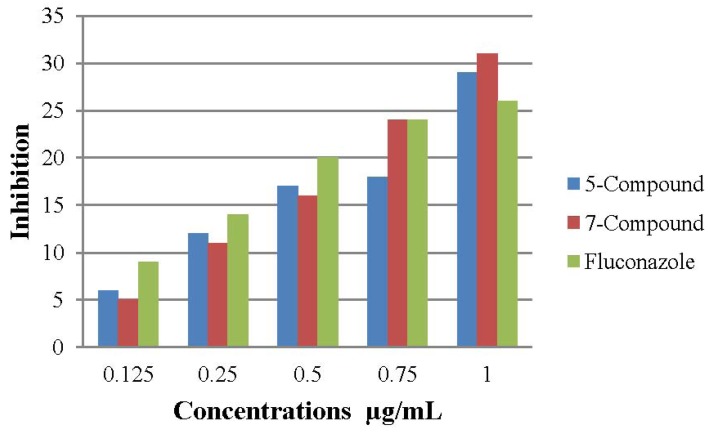
The effect of compounds **5** and **7** toward the tested organism *Candida albicans*.

*In vitro* antifungal screening effects of the investigated compounds **5** and **7**, were tested against some fungal spices (*Aspergillus niger* and *Candida albicans*). Compounds **5** and **7** showed good activities as antifungals compared to the antifungal ability of fluconazole, which was used as a standard (Figure 4 and Figure 5).

## 3. Experimental

### 3.1. General

The chemicals used during synthesis was supplied by Sigma-Aldrich. Purity of the compounds was checked on thin layer chromatography (TLC) plates (Silica gel G) in the solvent systems benzene-ethyl acetate-methanol (40:30:30, v/v/v) and toluene-acetone (75:25, v/v). The spots were located under 254 and 365 nm UV light. The IR spectra was obtained on a Thermo Scientific, NICOLET 6700 FTIR spectrometer (without out KBr or CsI pellets), at the Faculty of Engineering. The NMR spectra was obtained on a JEOL JNM-ECP 400, FT-NMR system at UKM, Faculty of Science Technology (FST), Chemistry Department. Elemental microanalysis, was carried out using a model 5500-Carlo Erba C.H.N elemental analyzer. A Gallenkamp M.F.B.600.010 F melting point apparatus was used to measure the melting points of all the prepared compounds.

#### 3.1.1. *Ethyl 2-((2-oxo-2H-chromen-4-yl)oxy)acetate* (**2**)

A suspension of 4-hydroxycoumarin (**1**, 6.17 mmol) in acetone (30 mL) was refluxed with ethyl bromoacetate (9.15 mmol) and K_2_CO_3_ (4.69 g, 33.91 mmol) for 12 h. After cooling, the mixture was evaporated to dryness and the residue was partitioned between CHCl_3_ (50 mL) and water (50 mL). The organic phase was dried (Na_2_SO_4_), filtered and evaporated to dryness. The residue was recrystallized from acetone; yield 92%; m.p. 99.0 °C; ^1^H-NMR (CDCl_3_): δ 3.22 (t, 3H, CH_3_), δ 3.85 (m, 2H, CH_2_), δ 4.91 (s, 2H) and δ 5.20, *δ* 5.250, δ 5.272 (s, 2H) for CH_2_), δ 5.78 (s, 1H) for -C=C-H), δ 7.291, δ 7.478, δ 7.80 (s, 1H) for aromatic ring); ^13^C-NMR: 167.2; 165.1; 163.4, 155.9; 134.2; 121.8; 121.1; 119.0; 113.8; 100.9; 65.3; 54.7; 22.12; IR: 2987.3 cm^−1^ (C-H, aliphatic), 3089.5 cm^−1^ (C-H, aromatic), 1759.3 cm^−1^ (C=O, lactone), 1717.6 cm^−1^ (C=O, ester), 1629.2 cm^−1 ^ (C=C, alkene), 1577.6 cm^−1^ (C=C, aromatic).

#### 3.1.2. *2-(2-Oxo-2H-chromen-4-yloxy)acetohydrazide* (**3**)

A solution of compound **2** (10 mmol) in ethanol (25 mL) was refluxed with hydrazine hydrate (15 mmol) for 4 h. After concentrating the reaction mixture a solid mass separated out and recrystallized using ethanol, yield 51%, m.p. 228 °C; ^1^H-NMR (CDCl_3_): δ 4.48 (s, 2H, CH_2_), δ 4.65 (s, 2H, NH_2_), δ 8.89 (s, 1H, NH), δ 4.92 (s, 2H) and *δ* 5.210 (s, 2H) for (O-CH_2_), δ 5.72 (s, 1H) for (-C=C-H), δ 7.410, δ 7.521, δ 8.10 (s, 1H) for aromatic ring; IR: 3297.3, 3211 cm^−1^ (N-H), 2906.0 cm^−1^ (C-H, aliphatic), 3072.7 cm^−1^ (C-H, aromatic), 1711.5 cm^−1^ (C=O), 1671.2 cm^−1^ (C=O, amide); Anal. Calc. for C_11_H_10_N_2_O_4_: C 56.41%, H 4.30%, N 11.96%. Found: C 57.13% H 4.01%, N 10.52%.

#### 3.1.3. *2-(2-(2-Oxo-2H-chromen-4-yloxy)acetyl)-N-phenylhydrazinecarbothioamide* (**4**)

A mixture of hydrazide **3 ** (2 mmol) and phenyl isothiocyanate (2 mmol) in ethanol (15 mL) was refluxed for 12 h. The reaction mixture was cooled and the separated product was filtered off, dried and recrystallized, using ethanol, yield 88%, m.p. 189–192 °C; ^1^H-NMR (CDCl_3_): δ 4.48 (s, 2H, CH_2_), δ 5.12 (s, 2H, NH_2_), δ 8.64 (s, 1H, NH), δ 4.80 (s, 2H) and *δ* 5.35 (s, 2H) for (O-CH_2_), δ 5.71 (s, 1H) for (-C=C-H), δ 7.41, δ 7.32, δ 7.92 (s, 1H) for aromatic ring; IR: 3368.9, 3371, 3350, 3262 cm^−1^ (N-H), 2929.0 cm^−1^ (C-H, aliphatic), 3074.6 cm^−1^ (C-H, aromatic), 1760.5 cm^−1^ (C=O, lactone), 1686.5 cm^−1^ (C=O), 1276.7 (C=S); Anal. Calc. for C_15_H_18_N_3_O_4_S: C 58.53%, H 4.09%, N 11.38%. Found: C 57.98% H 4.36%, N 10.93%.

#### 3.1.4. *4-((5-Mercapto-4-phenyl-4H-1,2,4-triazol-3-yl)methoxy)-2H-chromen-2-one* (**5**)

Compound **4** (0.5 mmol) was added to 8% NaOH solution (4 mL) and the reaction mixture was heated under reflux for 4 h. After cooling, the solution was acidified with a diluted solution of HCl. Crude product was precipitated, filtered off and washed with water. The solid thus separated was dried and recrystallized using chloroform-ether (1:1, v/v), yield 55%, m.p. 247–251 °C; ^1^H-NMR (CDCl_3_): δ 4.77 and 5.40 (d, 2H, OCH_2_), δ 5.81 (s, 1H) for (-C=C-H), δ 7.221–7.71 (m, 1H) for aromatic ring; IR: 3408 cm^−1^ (N-H), 2892.3 cm^−1^ (C-H, aliphatic), 3076.3 cm^−1^ (C-H, aromatic), 1758.29 cm^−1^ (C=O, lactone), 1577.8 cm^−1^ (C=N), 1634.2 cm^−1^ (C=C) and 1240 cm^−1^ (C=S). Anal Calc.for C_18_H_13_N_3_O_3_S: C 61.53%, H 3.73%, N 11.96%. Found: C 60.91% H .3.98%, N 11.14%.

#### 3.1.5. *4-((5-(Phenylamino)-1,3,4-thiadiazol-2-yl)methoxy)-2H-chromen-2-one* (**7**)

A mixture of compound **4** (0.15 mmol) and concentrated H_2_SO_4_ (5 mL) was stirred in an ice bath for 5 h and then at room temperature for another 5 h. The reaction mixture was neutralized with a diluted solution of ammonium hydroxide, in an ice bath. The precipitate was filtered off, washed with water, dried and recrystallized. using chloroform-ether (1:1, v/v), yield 65%, m.p. 240–242 °C; ^1^H-NMR (CDCl_3_): δ 4.96 and 5.35 (d, 2H, OCH_2_), δ 5.80 (s, 1H) for (-C=C-H), δ 7.21–8.02 (m, 1H) for aromatic ring; IR: 2900.1 cm^−1^ (C-H, aliphatic), 3079.7 cm^−1^ (C-H, aromatic), 1761.7 cm^−1^ (C=O, lactone), 1626.0 cm^−1^ (C=C), 1599.7 cm^−1^ (C=N) cm^−1^ Anal. Calc. for C_18_H_13_N_3_O_3_S: C 61.53%, H 3.73%, N 11.96%. Found: C 61.01% H 4.02%, N 11.13%.

### 3.2. DFT

Gaussian 03, Revision C.01 was used for the calculation of ground-state geometry that was optimized to a local minimum without any symmetry restrictions using the 3-21G basis set. The Becke three-parameter hybrid (B3) exchange functional in combination with the Lee-Yang-Parr (LYP) correction functional (B3LYP) was used for all geometry optimizations, thermodynamic functions at standard conditions (temperature = 298.150 Kelvin, and pressure = 1.0 Atm), High Occupied Molecular Orbital (HOMO) and Low Unoccupied Molecular Orbital (LUMO) distribution, and some physical properties for all molecules.

### 3.3. Evaluation of Antifungal Assay

Antifungal activity [25,26], was determined based on the growth inhibition rates of the mycelia of strains (*Aspergillus niger* and *Candida albicans*) in Potato Dextrose Broth medium (PDB). Under aseptic conditions, one mL of spore suspension (5 × 106 cfu/mL) of tested fungi was added to 50 mL PDB medium in a 100 mL Erlenmeyer flask. Appropriate volumes of tested compounds **5** and **7** were added to produce concentrations ranging from 10 to 100 μg mL^−1^. Flasks were incubated at 27 ± 1 °C in the dark for 5 days and then the mycelium was collected on filter papers. The filter papers were dried to constant weight and the level of inhibition, relative to the control flasks was calculated from the following formula:



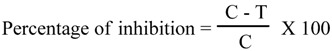



where T = weight of mycelium from test flasks and C = weight of mycelium from control flasks.

## 4. Conclusions

In this study, the compounds **2**–**7** were synthesized, and characterized using various spectroscopic methods and elemental analysis. The synthesized compounds **5** and **7** were studied theoretically and their atomic charges, heat of formation and stereochemistry were estimated, and it was found that they are not planar. The synthesized compounds **5** and **7** were tested for antifungal activities and indicated significant antifungal activities as compared with fluconazole.
